# Management of Post-stroke Shoulder Subluxation Using Sling Suspension-Based Active Shoulder Exercises: A Case Report

**DOI:** 10.7759/cureus.111023

**Published:** 2026-06-17

**Authors:** Mukta T Killedar, Suraj B Kanase

**Affiliations:** 1 Department of Neurosciences, Krishna Vishwa Vidyapeeth (Deemed to be University), Krishna College of Physiotherapy, Karad, IND

**Keywords:** case report, hemiparesis, neurorehabilitation, post-stroke shoulder subluxation, shoulder rehabilitation, sling suspension-based active shoulder exercises

## Abstract

Stroke-related shoulder subluxation is a common musculoskeletal complication that may interfere with upper limb rehabilitation and negatively affect recovery. Although previous studies have reported beneficial effects of sling suspension-based exercise programs for post-stroke shoulder subluxation, limited information is available regarding their detailed clinical application, treatment progression, and individual patient response.

This case report describes the outcome of a physiotherapy intervention in a 45-year-old male with left hemiparesis following a right-sided intraparenchymal hemorrhage with intraventricular extension. The patient was evaluated four months after the neurological event and presented with shoulder pain, increased muscle tone, restricted shoulder mobility, and inferior shoulder subluxation. Shoulder subluxation was assessed using a standardized tape measurement of the distance between the acromion process and the humeral head, which was 18 mm at baseline. A four-week rehabilitation program was implemented, comprising sling suspension-based active shoulder exercises, scapular stabilization exercises, and weight-bearing activities. The intervention was progressively advanced according to the patient's tolerance and clinical response. Therapy sessions were conducted for 30 minutes per day, five days per week. Following the intervention, the distance between the acromion process and the humeral head decreased from 18 mm to 11 mm, indicating improved shoulder alignment and reduced shoulder subluxation. Improvements were also observed in shoulder pain, muscle tone, active shoulder range of motion, and functional use of the affected upper extremity.

These findings suggest that a multimodal rehabilitation program incorporating sling suspension-based active shoulder exercises may be associated with improved shoulder alignment and favorable clinical outcomes in individuals with post-stroke shoulder subluxation. However, the specific contribution of each intervention component cannot be determined from a single case report. Further studies involving larger samples and controlled study designs are required to establish the effectiveness and generalizability of these findings.

## Introduction

Stroke frequently results in a range of neuromuscular and musculoskeletal impairments that can compromise upper limb recovery. Among these complications, shoulder subluxation is commonly observed in individuals with hemiparesis and represents a significant challenge during rehabilitation. The condition is characterized by inferior displacement of the humeral head relative to the glenoid fossa and is commonly attributed to weakness or impaired activation of the muscles responsible for maintaining glenohumeral stability. Shoulder subluxation often develops during the early flaccid stage following stroke and may persist if adequate motor recovery is not achieved. Previous reports have indicated that a substantial proportion of individuals with post-stroke hemiparesis experience shoulder subluxation during the course of recovery [1,2].

Shoulder subluxation can affect rehabilitation in multiple ways, beyond simply altering joint structure. Disruption of normal shoulder biomechanics can place excessive stress on periarticular tissues, contributing to pain, restricted range of motion, impaired upper limb function, and reduced use of the affected extremity. Furthermore, prolonged malalignment of the glenohumeral joint has been associated with secondary complications such as soft tissue injury, impingement syndromes, and adhesive capsulitis, all of which may hinder functional recovery and independence [3-5].

A wide range of therapeutic approaches has been investigated for the management of shoulder subluxation after stroke. Commonly used interventions include supportive slings, taping techniques, electrical stimulation, positioning strategies, and exercise-based rehabilitation programs. Although these interventions are frequently incorporated into clinical practice, current evidence suggests variable effectiveness, and consensus regarding the most effective treatment approach remains lacking. Consequently, there is continued interest in rehabilitation strategies that facilitate active muscular participation while simultaneously improving shoulder alignment and stability [1,6-13].

Sling suspension therapy has gained increasing attention as an exercise-based rehabilitation technique for individuals with neurological and musculoskeletal disorders. By providing adjustable support and partially unloading the limb from gravitational forces, sling systems enable controlled movement and facilitate activation of weakened musculature. Such characteristics may be particularly advantageous for individuals with impaired motor control following stroke. Previous studies have reported positive effects of sling-based exercise programs on balance, upper limb performance, and shoulder alignment in stroke populations. Jung and Choi reported improvements in shoulder subluxation, proprioception, and upper extremity function following sling suspension-based active shoulder exercises in individuals with acute stroke [14]. Similarly, Kim et al. demonstrated reductions in shoulder subluxation together with improvements in pain, muscle strength, and upper limb function among individuals with subacute stroke who participated in sling suspension-based exercise programs [15]. Additional evidence has demonstrated the beneficial effects of elastic dynamic sling interventions on shoulder alignment and upper limb recovery after stroke [16]. Emerging evidence therefore suggests that active shoulder exercises performed within a sling suspension system may reduce shoulder subluxation and improve glenohumeral alignment during the subacute stage of recovery [11-16].

Maintenance of normal glenohumeral alignment depends not only on rotator cuff and deltoid muscle activity but also on appropriate scapular control, muscle tone regulation, and coordinated scapulohumeral rhythm. Following a stroke, impairments in these mechanisms may contribute to altered shoulder biomechanics, pain, restricted movement, and the development of shoulder subluxation. By facilitating supported active movement and activation of shoulder stabilizing musculature, sling suspension-based exercise programs may help address some of these impairments and potentially contribute to improvements in shoulder alignment and function. Therefore, outcome measures assessing shoulder subluxation, pain, muscle tone, and range of motion were selected to evaluate clinical changes associated with the intervention.

Although these studies provide encouraging evidence regarding the potential benefits of sling-based rehabilitation approaches, most reports have focused primarily on group-level outcomes. Detailed descriptions of clinical implementation, treatment progression, clinical reasoning, and patient-specific responses remain limited. Nevertheless, detailed descriptions of the clinical application and individual patient response to sling suspension-based active shoulder exercise programs for post-stroke shoulder subluxation remain scarce. Therefore, this case report presents the application of sling suspension-based active shoulder joint exercises for the management of shoulder subluxation in an individual with subacute stroke and describes changes in shoulder alignment, pain, muscle tone, and shoulder mobility following the intervention.

## Case presentation

A 45-year-old right-hand-dominant male presented to the neurosciences outpatient department with complaints of left shoulder pain, weakness, and difficulty performing activities requiring functional use of the affected upper limb. The patient reported difficulty performing activities requiring reaching, lifting, and functional use of the affected upper extremity because of pain, weakness, and impaired shoulder control. Four months prior to presentation, he had experienced a right-sided intracerebral hemorrhage with intraventricular extension, resulting in left hemiparesis. Following acute medical management, the patient continued to experience persistent shoulder subluxation and pain that limited functional use of the affected upper extremity. He had no history of previous stroke or diabetes mellitus. Hypertension was diagnosed following the cerebrovascular event and was being managed medically. The patient had not undergone any structured physiotherapy rehabilitation or used any shoulder support device before enrollment in the intervention program. Following discharge from acute medical care, the patient continued to experience persistent shoulder pain, subluxation, and impaired upper limb function with minimal perceived improvement prior to commencement of the rehabilitation program.

Written informed consent for publication of this case report was obtained from the patient.

Neuroimaging demonstrated a right thalamocapsular and basal ganglia intracerebral hemorrhage with intraventricular extension, accompanied by perilesional edema, mass effect, and approximately 3.4 mm of leftward midline shift. Representative magnetic resonance imaging (MRI) findings are shown in Figure 1.

**Figure 1:**
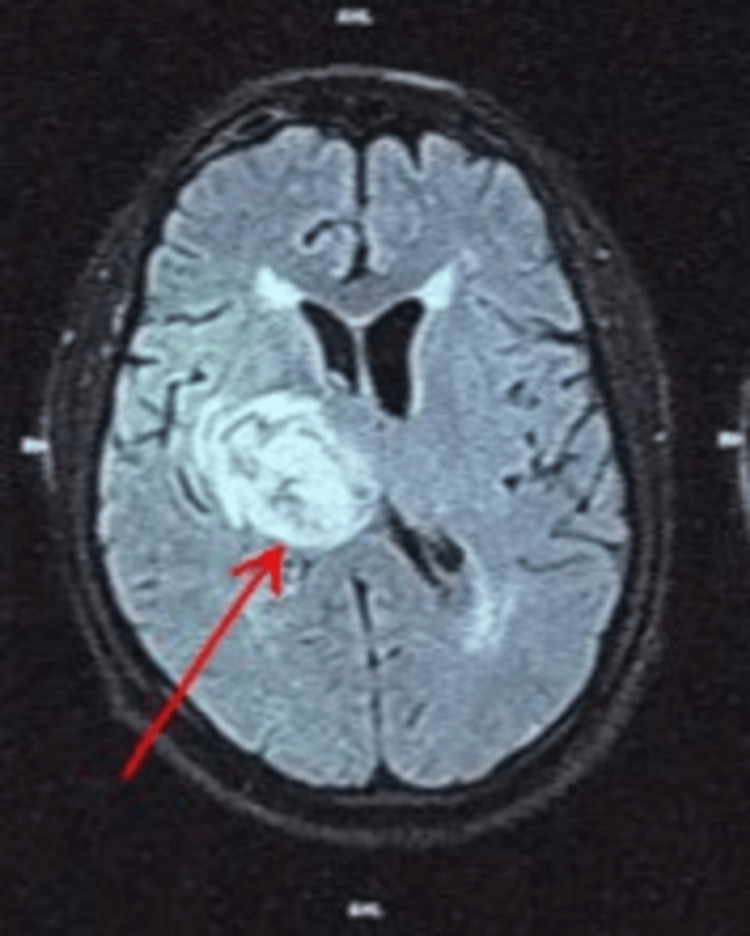
Axial MRI brain images demonstrating a right thalamocapsular and basal ganglia intracerebral hemorrhage with intraventricular extension

Shoulder pain was assessed using the numeric pain rating scale (NPRS) [17], muscle tone was evaluated using the modified Ashworth scale (MAS) [18], and shoulder range of motion was measured using a universal goniometer. Physical examination revealed visible inferior subluxation of the left shoulder. The patient reported shoulder pain rated 7/10 on the NPRS. The pain was localized to the affected shoulder region and was associated with difficulty performing activities requiring reaching, lifting, and functional use of the affected upper extremity. Pain was minimal at rest but increased during active movement, passive movement, and weight-bearing activities involving the affected upper limb. No night pain was reported. Clinical examination did not reveal findings strongly suggestive of adhesive capsulitis, rotator cuff pathology, complex regional pain syndrome, or central post-stroke pain. The clinical presentation was therefore considered consistent with shoulder pain associated with post-stroke shoulder subluxation and altered shoulder biomechanics.

Neurological examination revealed a flexor synergy pattern in the affected upper limb and an extensor synergy pattern in the affected lower limb. Superficial sensation, deep sensation, and combined cortical sensations were intact on clinical examination.

Muscle tone was assessed using the modified Ashworth scale (MAS) during passive shoulder flexion, extension, abduction, adduction, internal rotation, and external rotation. The overall muscle tone of the affected shoulder was graded as 2 on the MAS. Active range of motion of the left shoulder was restricted in all planes, with flexion of 70°, extension of 15°, abduction of 45°, external rotation of 10°, internal rotation of 35°, and horizontal abduction of 15°.

Shoulder subluxation was assessed using a standardized tape measurement technique by measuring the vertical distance between the lateral border of the acromion process and the superior aspect of the humeral head. Measurements were obtained with the patient in a supported sitting position. The same evaluator performed all measurements using the same procedure at baseline and follow-up. The baseline measurement was 18 mm, indicating significant inferior displacement of the humeral head. All outcome assessments were performed before the therapy session on the respective assessment day.

The detailed components of the sling suspension-based active shoulder exercise program are presented in Table 1.

**Table 1:** Description of the sling suspension-based active shoulder exercise intervention protocol

Intervention component	Exercise	Patient position	Therapist position and handling	Exercise performance	Dosage
Sling suspension-based active shoulder exercises	General sling setup	The affected upper limb was supported through straps attached to the distal forearm (just proximal to the wrist joint) and hand region (metacarpal level). In selected cases, an additional strap was used around the mid-forearm to improve stability and alignment. The sling height was adjusted to partially unload the upper limb against gravity while maintaining a functional, pain-free shoulder position and allowing free movement in all planes without traction stress at the glenohumeral joint.	The therapist stood on the affected side throughout the intervention and continuously monitored scapular position, joint alignment, and movement quality. Manual facilitation was provided at the scapula (proximal control) and forearm/wrist region (distal control) when required.	Exercises were performed within a pain-free range with emphasis on scapulohumeral rhythm, smooth motor control, and prevention of compensatory trunk movements.	—
Sling suspension-based active shoulder exercises	Shoulder flexion-extension	The patient lay in a side-lying position on the non-affected side with the affected upper limb supported in the sling at the distal forearm and hand region, allowing unrestricted sagittal plane movement.	The therapist stood at shoulder level on the affected side, stabilizing the scapula and guiding the forearm when required.	The patient actively performed shoulder flexion by moving the arm forward and upward, followed by controlled extension backward. The sling reduced gravitational load and facilitated smooth movement initiation.	20 repetitions
Sling suspension-based active shoulder exercises	Shoulder abduction-adduction	The patient lay in a supine position with the affected upper limb supported in the sling at the distal forearm and hand region and positioned close to the body at rest. The sling was adjusted to allow free frontal plane movement without shoulder stress.	The therapist stabilized the scapula and assisted with distal control when required.	The patient actively moved the arm away from the midline into abduction and returned it to adduction in a controlled manner.	20 repetitions
Sling suspension-based active shoulder exercises	Shoulder internal-external rotation	The patient sat upright with the affected upper limb supported in the sling at the distal forearm and hand region, with the elbow maintained at 90° flexion and the shoulder in neutral position.	The therapist monitored from the affected side and stabilized the elbow or forearm when required.	The patient performed external rotation by moving the forearm outward and internal rotation by bringing it toward the abdomen, enabling isolated glenohumeral movement.	20 repetitions
Sling suspension-based active shoulder exercises	Shoulder horizontal abduction-adduction	The patient sat upright with the trunk in neutral alignment and the affected upper limb supported in the sling at the distal forearm and hand region, adjusted at shoulder height.	The therapist stood slightly behind the affected side to monitor scapular control and trunk stability.	The patient actively moved the arm horizontally away from the midline and returned it to the starting position in a controlled manner.	20 repetitions
Weight-bearing exercises	Quadruped anterior-posterior and lateral weight shifts	The patient assumed a quadruped position with the affected upper limb in full elbow extension, wrist in neutral, and palm placed firmly on the support surface. Proper alignment was maintained through the wrist, elbow, and shoulder, with the scapula in a neutral position.	The therapist stood on the affected side to continuously monitor scapular stability, trunk alignment, and overall posture. Manual support was provided at the scapula and/or pelvis when required to prevent shoulder collapse, scapular winging, or trunk asymmetry. Verbal and tactile cues were used to maintain proper weight distribution.	The patient performed slow, controlled anterior-posterior and lateral weight shifts within a pain-free range while maintaining stability and alignment.	20 repetitions
Weight-bearing exercises	Bedside sitting weight bearing through the affected upper limb	The patient sat upright at the edge of the plinth with feet supported on the floor. The affected upper limb was placed on the plinth beside the hip with the elbow extended, wrist in neutral, and palm in contact with the surface.	The therapist stood on the affected side to ensure optimal trunk alignment and scapular control. Manual contact was provided at the scapula and trunk to prevent lateral trunk lean, shoulder hiking, or loss of midline orientation. Assistance was adjusted according to patient stability.	The patient performed controlled weight shifts onto the affected upper limb while maintaining upright posture and scapular stability.	20 repetitions
Scapular stabilization exercises	Shoulder shrugs (elevation-depression)	The patient was positioned in an upright sitting posture on a plinth with feet supported on the floor and trunk maintained in neutral alignment. The affected upper limb was relaxed alongside the body.	The therapist stood on the affected side to monitor scapular movement and postural alignment.	The patient actively performed shoulder elevation by shrugging both shoulders upward followed by controlled relaxation into the neutral position in a slow and controlled manner within a pain-free range.	20 repetitions
Scapular stabilization exercises	Scapular protraction-retraction	The patient was seated upright with the trunk in neutral alignment and shoulders relaxed.	The therapist stood on or slightly behind the affected side to observe scapular mechanics and ensure correct movement patterns. Manual guidance was provided along the medial and lateral borders of the scapula when required to facilitate proper protraction and retraction while minimizing trunk compensation.	The patient actively performed scapular protraction by moving the shoulders forward and retraction by drawing them backward in a controlled and smooth manner within a pain-free range.	20 repetitions

The sling suspension-based exercise program included a series of gravity-minimized, active shoulder movements performed in different positions. The side-lying sling suspension-based active shoulder flexion-extension exercise was performed with the patient positioned in side-lying, allowing controlled flexion and extension movements (Figure 2). 

**Figure 2:**
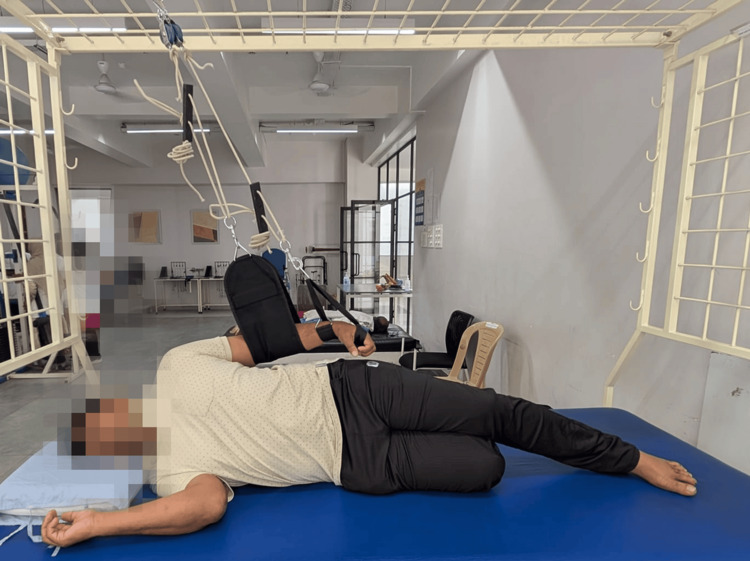
Sling suspension-based active shoulder flexion and extension exercises performed in side-lying position with partial unloading of the affected upper limb

The supine sling suspension-based active shoulder abduction-adduction exercise enabled active abduction and adduction movements in a gravity-minimized plane (Figure 3).

**Figure 3:**
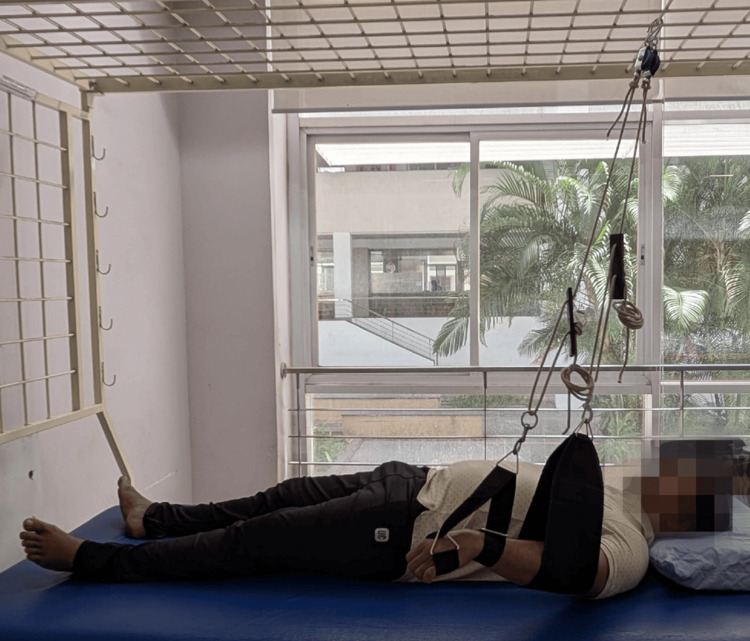
Sling suspension-based active shoulder abduction and adduction exercises performed in the supine position with partial unloading of the affected upper limb

For horizontal adduction and abduction, the sling suspension system was adjusted to provide partial unloading of the affected upper limb, thereby reducing gravitational load while still permitting active muscle recruitment. The patient was positioned in a sitting position with the shoulder at approximately 90° of flexion, facilitating active horizontal adduction (Figure 4). 

**Figure 4:**
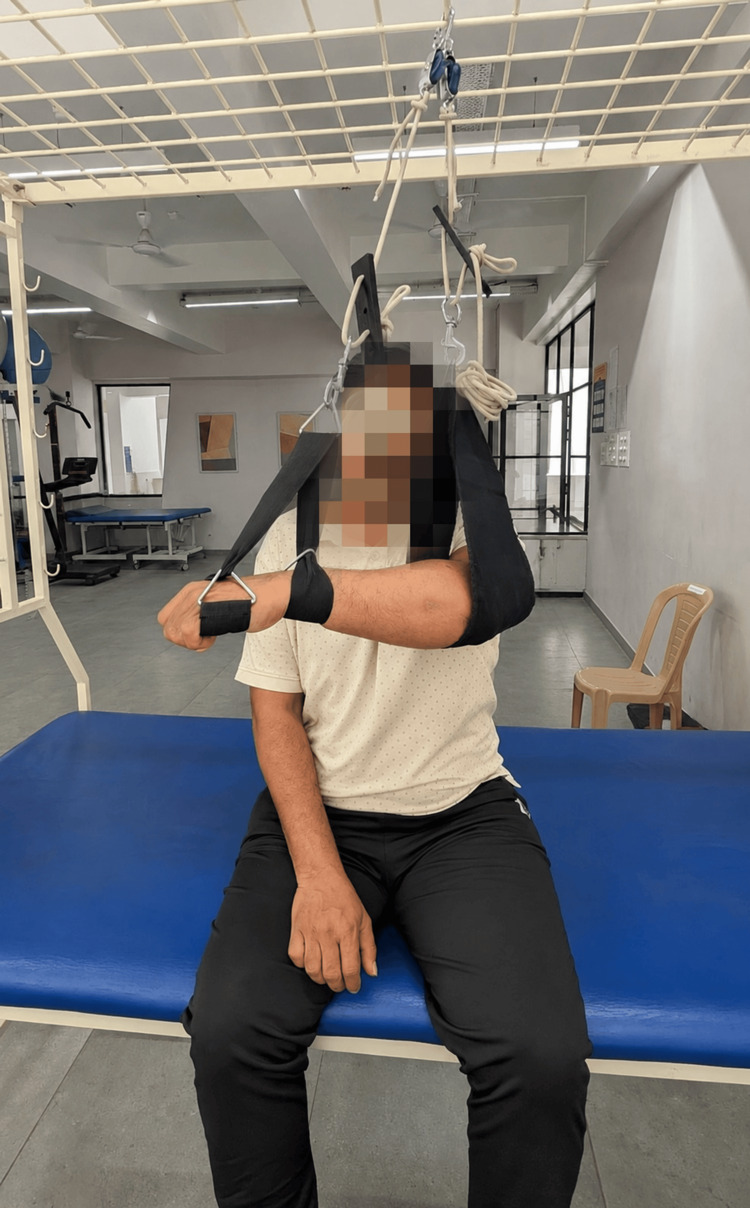
Sling suspension-based active horizontal shoulder adduction exercise in sitting position with partial unloading of the affected upper limb

For horizontal abduction, the patient was positioned in a sitting position with the shoulder at approximately 90° of flexion, facilitating active horizontal abduction (Figure 5). 

**Figure 5:**
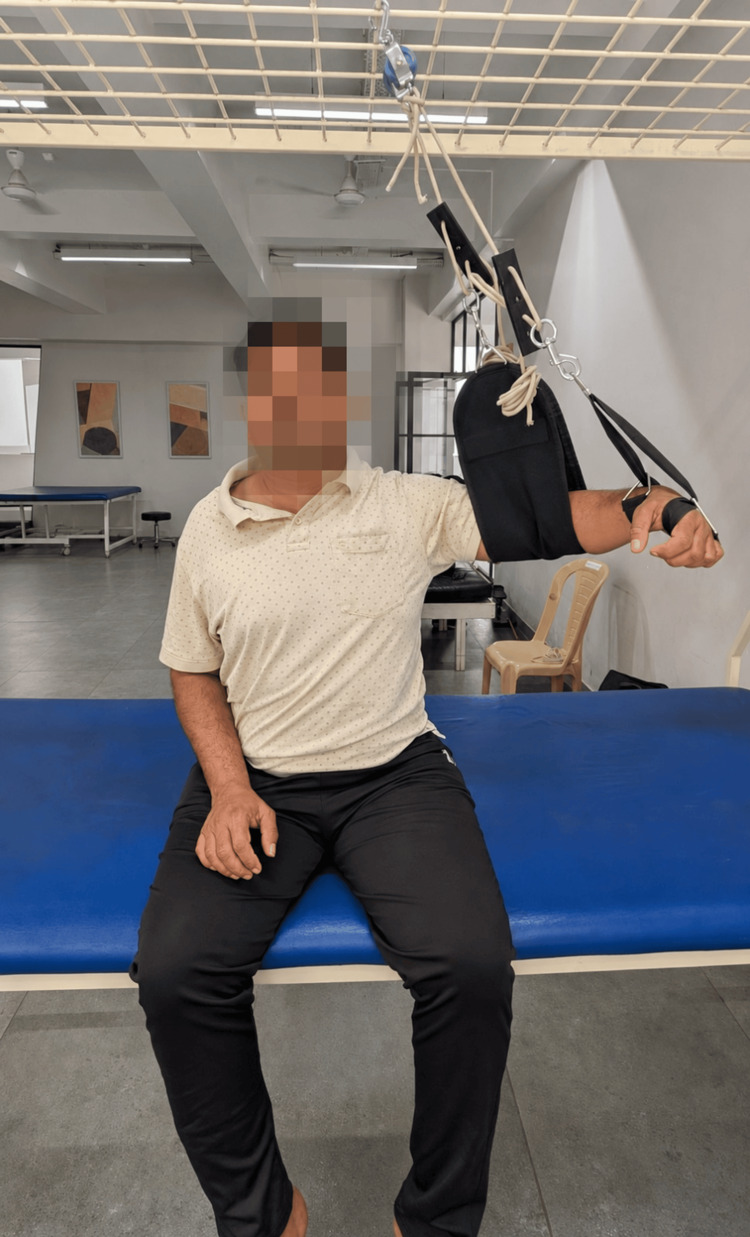
Sling suspension-based active horizontal shoulder abduction exercise in sitting position with partial unloading of the affected upper limb

Rotational movements were also performed in a sitting position with the shoulder in a neutral position and the elbow flexed. Active internal rotation of the shoulder was performed in a sitting position with the shoulder maintained in neutral and the elbow flexed (Figure 6). 

**Figure 6:**
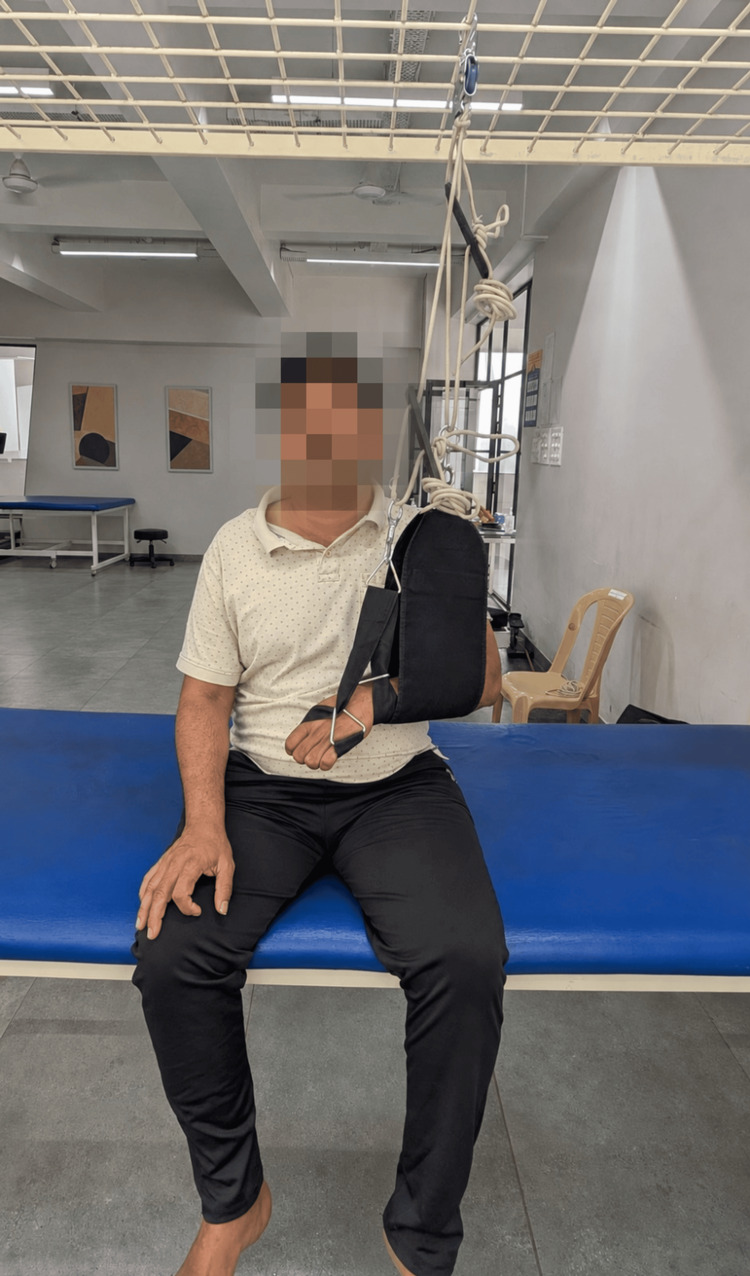
Sling suspension-based active shoulder internal rotation exercise performed in sitting position with partial unloading of the affected upper limb

Active external rotation of the shoulder was performed in a sitting position with the shoulder maintained in neutral and the elbow flexed (Figure 7). 

**Figure 7:**
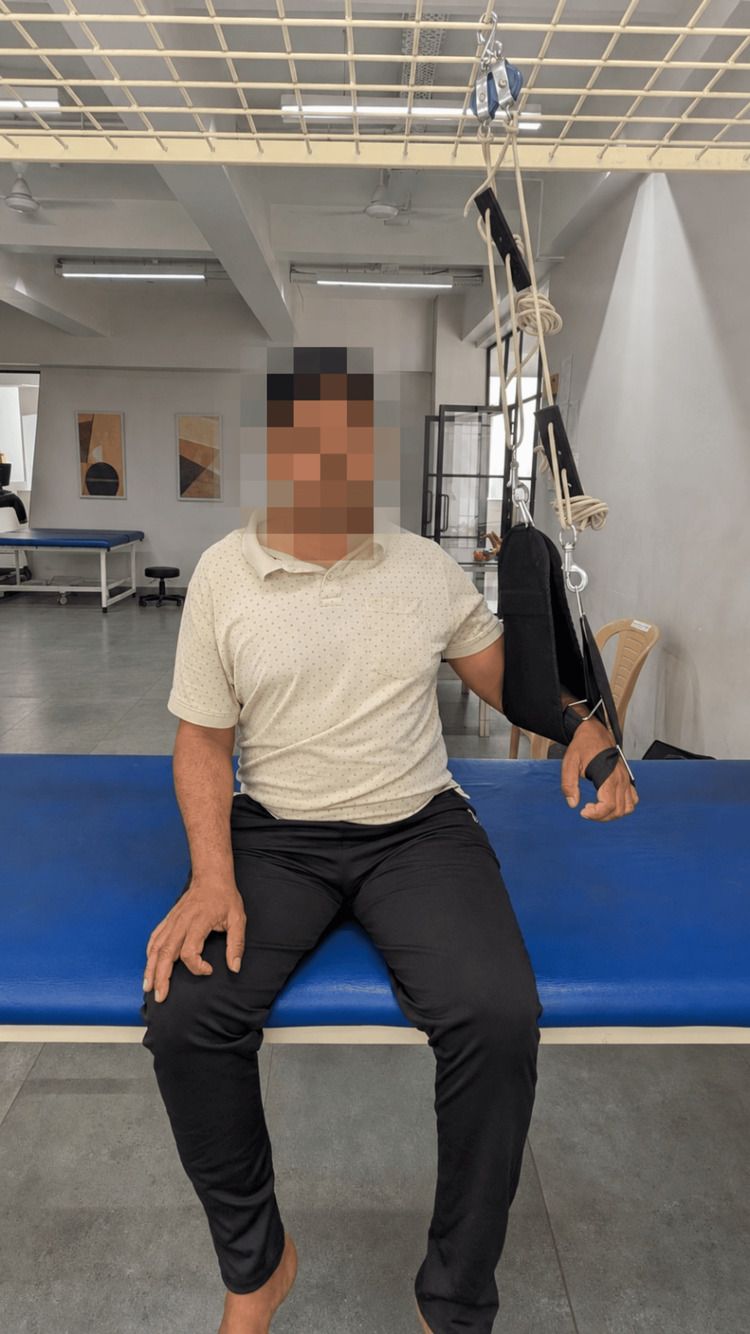
Sling suspension-based active shoulder external rotation exercise performed in sitting position with partial unloading of the affected upper limb

Intervention

The intervention was selected because the patient presented with shoulder subluxation, pain, restricted active movement, and impaired shoulder control following a stroke. Sling suspension-based exercises were chosen to provide partial support to the affected upper limb, thereby reducing the influence of gravity while facilitating active movement. This approach was intended to promote activation of the shoulder stabilizing musculature and improve glenohumeral alignment during functional movement.

The patient underwent a four-week physiotherapy rehabilitation program consisting of sling suspension-based active shoulder exercises, scapular stabilization exercises, and weight-bearing activities. The detailed components of the intervention program are presented in Table 1. Treatment sessions were conducted for 30 minutes per day, five days per week for four weeks under the supervision of a physiotherapist. A rest period of approximately 30 seconds was provided between exercise sets.

At the beginning of rehabilitation, the patient demonstrated limited active shoulder control and experienced pain during movement. Therefore, exercises were initially performed within a restricted pain-free range while emphasizing proper scapular alignment and movement quality. As pain levels decreased and shoulder stability improved, the patient was encouraged to perform a greater range of active movement with reduced therapist assistance. During the later stages of the program, the patient was able to perform exercises through a larger range of motion with improved movement control and reduced compensatory shoulder movements compared with the initial sessions. Progression of the exercise program was guided by the patient's tolerance and clinical response throughout the intervention period. Therapist assistance was gradually reduced from maximal to minimal support, patient participation was progressively increased, and the available range of motion was increased as tolerated while maintaining all movements within a pain-free range.

During the intervention period, the patient did not receive analgesic medications, oral antispasticity medications, botulinum toxin injections, occupational therapy, additional physiotherapy interventions, or shoulder support devices. Therefore, no concurrent rehabilitation interventions specifically targeting shoulder subluxation were administered during the treatment period.

The patient completed the intervention program without adverse events and adhered well to the prescribed treatment sessions.

Outcomes

Following completion of the intervention, reassessment demonstrated a reduction in the distance between the acromion process and the humeral head from 18 mm to 11 mm, indicating improved shoulder alignment and a reduction in shoulder subluxation. Shoulder pain decreased from 7/10 to 3/10 on the NPRS. The patient also reported reduced pain during active movement, passive movement, and weight-bearing activities compared with baseline. In addition, the patient reported improved ability to perform activities requiring functional use of the affected upper extremity. The overall muscle tone of the affected shoulder improved from grade 2 to grade 1+ on the modified Ashworth scale (MAS). Improvements were also observed in active shoulder range of motion, with flexion increasing from 70° to 95°, extension from 15° to 20°, abduction from 45° to 60°, external rotation from 10° to 18°, internal rotation from 35° to 42°, and horizontal abduction from 15° to 25°. In addition, the patient demonstrated improved shoulder movement control and reduced compensatory shoulder movements during performance of the exercise program compared with the initial treatment sessions. Although scapular position and scapular dyskinesia were not formally assessed using standardized outcome measures, improved scapular control was observed clinically during upper limb movements following the intervention. Clinical findings before and after the intervention are summarized in Table 2.

**Table 2:** Clinical outcomes before and after intervention NPRS - numeric pain rating scale; MAS - modified Ashworth scale

Outcome measure	Pre-intervention	Post-intervention
Distance between acromion process and humeral head (mm)	18	11
Numeric pain rating scale (NPRS)	7/10	3/10
MAS - shoulder flexion	Grade 2	Grade 1+
MAS - shoulder extension	Grade 2	Grade 1+
MAS - shoulder abduction	Grade 2	Grade 1+
MAS - shoulder adduction	Grade 2	Grade 1+
MAS - shoulder internal rotation	Grade 2	Grade 1+
MAS - shoulder external rotation	Grade 2	Grade 1+
Shoulder flexion	70°	95°
Shoulder extension	15°	20°
Shoulder abduction	45°	60°
Shoulder external rotation	10°	18°
Shoulder internal rotation	35°	42°
Shoulder horizontal abduction	15°	25°

## Discussion

The present case report evaluated the effects of a four-week rehabilitation program incorporating sling suspension-based active shoulder exercises in an individual with post-stroke shoulder subluxation. Following the intervention, improvements were observed in shoulder alignment, pain intensity, muscle tone, and active shoulder mobility. The distance between the acromion process and humeral head decreased from 18 mm to 11 mm, accompanied by reductions in pain and muscle tone and increases in shoulder range of motion. These findings suggest that participation in a rehabilitation program incorporating sling suspension-based active shoulder exercises may be beneficial for individuals with shoulder subluxation during the subacute stage of stroke recovery.

Shoulder subluxation is a common complication following stroke and is frequently associated with pain, altered shoulder biomechanics, impaired upper limb performance, and delayed motor recovery [1-6]. Weakness of the shoulder stabilizing musculature may result in inferior displacement of the humeral head, thereby contributing to secondary musculoskeletal complications and restricting participation in rehabilitation [1,3,4]. Effective management of shoulder subluxation is therefore important to optimize upper limb recovery and functional outcomes after stroke [5,6].

The improvements observed in the present case are consistent with findings reported in previous studies investigating sling-based rehabilitation approaches. Jung and Choi demonstrated that active shoulder exercises performed using a sling suspension system improved shoulder subluxation, proprioception, and upper extremity function in individuals with acute stroke [14]. Similarly, Kim et al. reported that sling-suspension-based active shoulder exercises reduced shoulder subluxation while improving pain, muscle strength, and upper limb function among patients with subacute stroke [15]. Kim et al. further demonstrated the beneficial effects of elastic dynamic sling interventions on shoulder subluxation in individuals with subacute stroke [16]. Collectively, these findings support the role of sling-based exercise programs in the rehabilitation of post-stroke shoulder dysfunction.

Sling suspension systems provide partial unloading of the upper limb, reducing the effects of gravity. This support facilitates active movement within a controlled and supported environment [11]. This support may promote activation of weakened shoulder stabilizers and allow movement practice with improved control and reduced compensatory strategies. Repetitive active exercise may facilitate neuromuscular re-education, enhance motor control, and contribute to improved glenohumeral stability. Previous evidence suggests that sling-based exercise programs may improve neuromuscular activation, postural control, and motor performance in individuals with neurological impairments [11,12].

The reduction in shoulder pain observed in the present case may be related to improved shoulder alignment and stabilization. Previous studies have demonstrated a relationship between shoulder subluxation and post-stroke shoulder pain, suggesting that restoration of glenohumeral alignment may reduce mechanical stress on periarticular structures and improve movement efficiency [2-4,8]. The decrease in pain from 7/10 to 3/10 on the numeric pain rating scale suggests that improved shoulder stability may have contributed to greater comfort during movement and daily activities.

In addition to improvements in shoulder alignment and pain, clinically meaningful gains were observed in active shoulder range of motion. Improvements were evident across all assessed movement planes, including flexion, extension, abduction, external rotation, internal rotation, and horizontal abduction. These gains may be attributed to reduced pain, enhanced shoulder stability, and improved activation of the shoulder musculature during exercise. Furthermore, functional outcome measures were not included; therefore, the influence of the intervention on overall upper limb function and activities of daily living could not be established. Although improvements were observed in shoulder alignment, pain, muscle tone, and range of motion, these impairment-level changes do not necessarily translate into meaningful improvements in upper limb function, task performance, or independence in daily activities. Consequently, the clinical significance of the observed improvements should be interpreted with caution. Future studies should incorporate standardized functional outcome measures to determine whether improvements in shoulder impairments are accompanied by clinically relevant functional gains.

A reduction in muscle tone was also observed following the intervention, as reflected by the improvement in modified Ashworth scale scores from grade 2 to grade 1+. Although reduction of muscle tone was not the primary objective of treatment, repetitive active movement and improved neuromuscular control may have contributed to more normalized muscle activity of the shoulder musculature. Reduced muscle tone may also have facilitated greater shoulder mobility and improved movement quality during functional activities.

Changes in muscle tone may also have influenced shoulder alignment. Increased tone in specific shoulder muscle groups can affect glenohumeral positioning; therefore, the contribution of altered muscle tone to the observed reduction in shoulder subluxation cannot be excluded.

Scapular alignment may also have influenced the observed outcomes. Although scapular position and scapular dyskinesia were not formally assessed using a standardized clinical assessment tool before or after the intervention, improvements in scapular control and reduced compensatory shoulder movements were observed clinically during treatment sessions. Changes in scapular alignment may have contributed to improved glenohumeral positioning and the reduction in shoulder subluxation. Future studies should incorporate standardized assessments of scapular kinematics and scapular positioning to better understand their relationship with shoulder alignment and recovery following stroke.

The observed improvements should be interpreted as changes temporally associated with participation in a multimodal rehabilitation program that included sling suspension-based active shoulder exercises, scapular stabilization exercises, and weight-bearing activities rather than as evidence of a specific isolated effect of sling suspension exercises alone.

From a clinical perspective, this case highlights the potential value of incorporating sling suspension-based active shoulder exercises into rehabilitation programs for individuals with post-stroke shoulder subluxation. The intervention was feasible to administer, well-tolerated by the patient, and associated with favorable changes across multiple clinical outcomes. These observations are consistent with previous reports suggesting that active rehabilitation strategies may provide advantages over passive support approaches alone in the management of shoulder subluxation after stroke [6,7,9,10,19].

Although previous controlled studies have reported favorable outcomes associated with sling suspension-based active shoulder exercises for reducing shoulder subluxation after stroke [14-16], the present case report provides a detailed description of the clinical application of the intervention in an individual patient. The report highlights baseline clinical presentation, treatment progression, clinical reasoning, and patient-specific responses that may not be fully captured through group-level analyses. Such information may assist clinicians in translating research findings into individualized rehabilitation practice.

Several limitations should be acknowledged. As this report describes the outcome of a single individual, the findings cannot be generalized to all patients with post-stroke shoulder subluxation. The absence of a comparison condition limits the ability to determine whether the observed improvements were solely attributable to the intervention. Furthermore, the intervention consisted of a multimodal rehabilitation program that included sling suspension-based active shoulder exercises, scapular stabilization exercises, and weight-bearing activities; therefore, the specific contribution of each intervention component cannot be determined. Furthermore, functional outcome measures were not included; therefore, the influence of the intervention on overall upper limb function and activities of daily living could not be established. Scapular position and scapular dyskinesia were not formally assessed using standardized outcome measures, limiting interpretation of their potential contribution to the observed improvements in shoulder alignment. Long-term follow-up was not performed, preventing evaluation of the durability of treatment effects. Although the patient reported minimal improvement prior to initiation of rehabilitation, the potential influence of natural recovery cannot be completely excluded. Future studies involving larger samples and controlled study designs are warranted to further investigate the effectiveness of rehabilitation programs incorporating sling suspension-based active shoulder exercises for post-stroke shoulder subluxation.

## Conclusions

This case report demonstrated that a four-week multimodal rehabilitation program consisting of sling suspension-based active shoulder exercises, scapular stabilization exercises, and weight-bearing activities was temporally associated with improvements in shoulder alignment, pain, muscle tone, and shoulder range of motion in an individual with post-stroke shoulder subluxation. Following the intervention, the distance between the acromion process and humeral head decreased from 18 mm to 11 mm, indicating improved shoulder alignment and reduced shoulder subluxation. Clinically meaningful reductions in pain and muscle tone were also observed, accompanied by gains in active shoulder mobility. The intervention was well tolerated, and these findings suggest that a multimodal rehabilitation program incorporating sling suspension-based exercises may be beneficial for individuals with post-stroke shoulder subluxation. However, the specific contribution of each intervention component cannot be determined from a single case report. Further research involving larger samples and controlled study designs is required to confirm these findings and establish their generalizability.
